# A Clinical Case of Viral Keratitis

**DOI:** 10.7759/cureus.30311

**Published:** 2022-10-14

**Authors:** Devanshu Gupta, Sachin Daigavane

**Affiliations:** 1 Department of Ophthalmology, Jawaharlal Nehru Medical College, Datta Meghe Institute of Medical Sciences, Wardha, IND

**Keywords:** infection, antivirals, cornea, herpes simplex virus, viral keratitis

## Abstract

Keratitis is a pathological condition involving inflammation of the cornea. It can be an infectious or non-infectious disease. The causative organisms of keratitis are categorized as bacteria, viruses, fungi, or parasites. The viruses responsible for causing keratitis are herpes simplex virus (HSV), varicella-zoster virus, and adenoviruses. The clinical features of this infection may range from pain and redness of the eye to scarring of the cornea or blindness. We present the case of a 71-year-old elderly female patient suffering from viral (HSV) keratitis. She was referred to the department of ophthalmology with complaints of diminution of vision and watering in the right eye associated with pain and redness for one month, which was progressive and gradual in onset. On local examination, the surface of the cornea was irregular in the right eye, with the presence of old keratitis precipitates. Viral infection is the second leading cause of keratitis and is very common in the western world. Because the transmission is due to droplets and fomites, strict measures must be taken to prevent transmission. If anyone comes in contact, prophylactic antiviral therapy can be administered. The prognosis is favorable if adequately treated. It can lead to blindness if not treated on time.

## Introduction

Viral keratitis is one of the most widespread diseases. The most common etiology is type 1 herpes simplex virus (HSV). The other organisms responsible include varicella-zoster virus, Epstein-Barr virus, and cytomegalovirus. The disease, if left untreated, can lead to corneal perforation and, ultimately, blindness. It is usually asymptomatic but can be mildly symptomatic. Patients can present with conjunctivitis associated with redness, discomfort, and tenderness. Various investigations can be used to diagnose the condition. Treatment options include topical eyedrops and debridement along with antivirals. Immunosuppressed or immunocompromised individuals may display recurring diseases with pronounced severity [1].

## Case presentation

**Figure 1:**
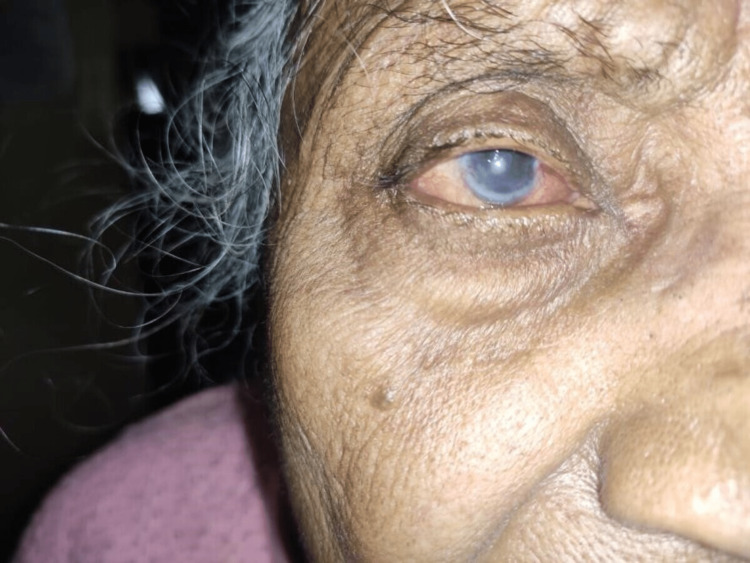
Involvement of the right eye showing conjunctival congestion, and corneal macular opacity present inferiorly.

A 71-year-old female patient came to the ophthalmology outpatient department (OPD) on October 21, 2021. She was a farmer by occupation who came with the chief complaints of gradual and progressive loss of vision in the right eye for 30 days, watering from the right eye envelope for one month which was associated with intermittent redness for one month, and pain in the right eye for 30 days. The patient appeared to be well until one month ago when she began to experience wetness and pain in her right eye, which was gradual at the beginning and progressive in nature. There was a positive history of photophobia. There were no complaints of sticky or mucopurulent discharge and colored halos. Diplopia was absent in both eyes. There was no recent history of trauma to the eyes. The patient had no history of cough, cold, or fever. She did not wear any contact lenses. There was no prolonged use of topical medications. There were no associated systemic complaints. The patient had no history of hypertension, diabetes, syphilis, tuberculosis, or bronchial asthma. There was no significant family history as well. She was cooperative and well-oriented to time, place, and person. She had an average build and was afebrile. Her pulse rate was 79 beats per minute, and her blood pressure was 124/78 mmHg. There was no pallor, clubbing, icterus, or lymphadenopathy on examination. On examination, the central nervous system and cardiovascular systems were within normal limits. The chest was clear bilaterally, and on per abdomen examination, she had no apparent distress. She provided a history of similar episodes, which occurred seven months ago, when she was diagnosed with herpetic neuralgia involving the right eye and forehead with a history of weakness on the right side of the forehead with rashes on the upper lid and forehead, for which she was put on tablet gabapentin, tablet Tryptomer, and tab Neurobion Forte for 15 days. The patient had a history of one more episode involving the right eye in the past seven months. A cataract extraction surgery was done in the right eye seven years ago and in the left eight years ago. Facial symmetry was present, head posture was normal, and forehead was normal. Visual acuity examined in the right-sided eye was CF1M, PL+PR accurate, and the left-sided eye visual acuity was 6/36 with pinhole 6/24P. Color vision, eyebrows, eyelids position, and movement were all normal in both eyes. Nasal pterygium was present in the bulbar conjunctiva, concretions and congestion were present on the lower palpebral conjunctiva of the right eye, whereas the conjunctiva on the left eye was normal. Old keratitic precipitation was present on the corneal surface of the right eye, and diffuse macular opacity was present from 4 o’clock to 8 o’clock. The left eye cornea was normal. The iris and pupil were normal, with irregular pupillary reflex in both eyes. Pseudopakia was present in both eyes, and the lacrimal apparatus was normal (Figure 1).

Therapeutic intervention

In ophthalmology, topical corticosteroids are widely used to suppress inflammation and are applied topically or systemically [2]. For viral keratitis, the patient was started on tablet acyclovir 400 mg three times a day, tablet pantoprazole 40 mg once a day, eyedrop carboxymethylcellulose two hourly, and eye ointment ganciclovir 0.15% three times a day. Antiviral treatment aimed to stop the viral infection, which was still active in the cornea to relieve the patient’s symptoms and restore normal function. Acyclovir halted the transcription of the virus, thereby limiting its replication. Corneal epithelial debridement or chemical cauterization removed or destroyed viral-inflamed cells of the corneal floor, and residual or continually shed virus was observed utilizing corneal epithelial regeneration without the recrudescent ocular infection [3]. The patient was kept on regular follow-ups. Symptomatic improvement was seen. Antiviral treatment was strictly monitored.

## Discussion

Corneal diseases are known to be the primary cause of loss of vision globally. Keratitis, a type of corneal disease, is defined as corneal inflammation. It can be characterized by corneal edema, cellular infiltration, and congestion in the cilia. Various organisms are responsible for causing keratitis, including bacteria, viruses, fungi, and protozoa. Primary HSV eye infection is contracted by direct contact via mucus membranes. This is usually subclinical but can present as transient unilateral blepharitis, follicular conjunctivitis, and occasional epithelial keratitis [4]. Topical antibiotics continue to be the remedy for bacterial keratitis, and a recent evaluation identified all usually prescribed topical antibiotics to be similarly effective [5]. Viruses are parasites that are intracellular and obligate in nature and cannot replicate through binary fission. Viruses are responsible for causing viral keratitis. This is a condition in which there is corneal inflammation which is both physiological and pathological. The incidence of viral corneal ulcers has been increasing due to the use of antibiotics to eliminate pathogenic flora. Most of the viruses tend to involve both the conjunctiva and cornea. Hence, the lesion constitutes viral keratoconjunctivitis. Viruses that are mainly responsible are HSV, varicella-zoster virus, and adenovirus. HSV keratitis is a common cause of ocular and visual morbidity [6]. It is among the major causes of corneal loss of vision. It is generally a unilateral disease, which means only one eye is affected in most cases. Viral keratitis occurs in either primary or recurrent forms. Primary keratitis is seen in young individuals but is rare. The recurrent infection consists of two stages, namely, acute and chronic. The causes of recurrent keratitis can be trauma, overexposure to ultraviolet light, and fever. The clinical features are watering of the eyes, irritation, photophobia, and loss of vision. There can be corneal ulcerations present at times. There are various lesions seen in this disease, one of them is a dendritic ulcer which is the most common lesion. It can be visualized by fluorescein staining, which indicates common branching, linear samples with feathery edges, and terminal bulbs at the edges. Other lesions caused are stellate epithelial keratitis, subepithelial lesions, disciform keratitis, and geographical ulcers. Microbial keratitis is a condition that affects people globally, with an estimated 1.5-2 million cases of corneal ulcers in underdeveloped nations [7]. The most common cause of viral hepatitis is HSV. HSV is a DNA virus, and it is of two types HSV1 and HSV2. HSV1 infection can be transmitted through kissing and coming in close contact with an already infected patient. HSV2 infection is acquired by the infected genitalia of the mother to the eyes of neonates. The ocular lesions occur in two forms, namely, primary and recurrent. The primary infection affects the conjunctiva causing acute follicular conjunctivitis. It also affects the cornea resulting in dendritic ulcers and skin lesions on the lid. HSV causes a spectrum of ocular diseases, but the most prominent are epithelial and stromal keratitis [8]. The lesion seen in recurrent infection is punctate epithelial keratitis, disciform keratitis, and geographical ulcers. The pathogenesis and severity of herpes simplex keratitis are largely determined by an interaction between viral genes encoded by the strain of HSV1 and the makeup of the host’s immune system [9]. The clinical manifestations of viral keratitis include mild fever, malaise, and non-suppurative lymphadenopathy, as mentioned earlier. Vesicular skin lesions involving the face, lids, periorbital region, and lid margins are also seen. Diagnosis is mainly made clinically. Lab investigations such as immunohistochemistry, immunofluorescence assay, and in situ hybridization are reliable for diagnosis. A slit lamp examination helps in the detection of the character and severity of keratitis. A thorough analysis of all presenting structures is also performed. The purpose of the treatment of this condition is to halt the active replication of the virus within the cornea and eliminate the inflammation from the cornea. Antiviral therapy is the choice of treatment as it increases epithelial healing and stops viral replication. Ganciclovir is a broad-spectrum antiviral agent [10]. Debridement of the epithelium is done to correct dendritic keratitis in which the infected epithelium is removed using a cotton tip applicator. Acyclovir can also be given orally to manage intense herpetic eye sickness, mainly in atopic individuals, immune regulatory drugs, together with cyclosporine A. Given the immune-mediated etiology of stromal illness, this is an intriguing alternative for dealing with HSV stromal keratitis. Surgical intervention is also done sometimes, wherein penetrating keratoplasty is performed.

## Conclusions

This was a case of viral keratitis in which an elderly female patient presented with diminution of vision and watering of eyes. The patient was immediately started on antiviral treatment with acyclovir. Viral keratitis is a diagnosis of high importance and should be investigated on time. Treatment related to the cause should be started. Our patient showed considerable improvement on follow-up. Therefore, this was a case worth reporting.
